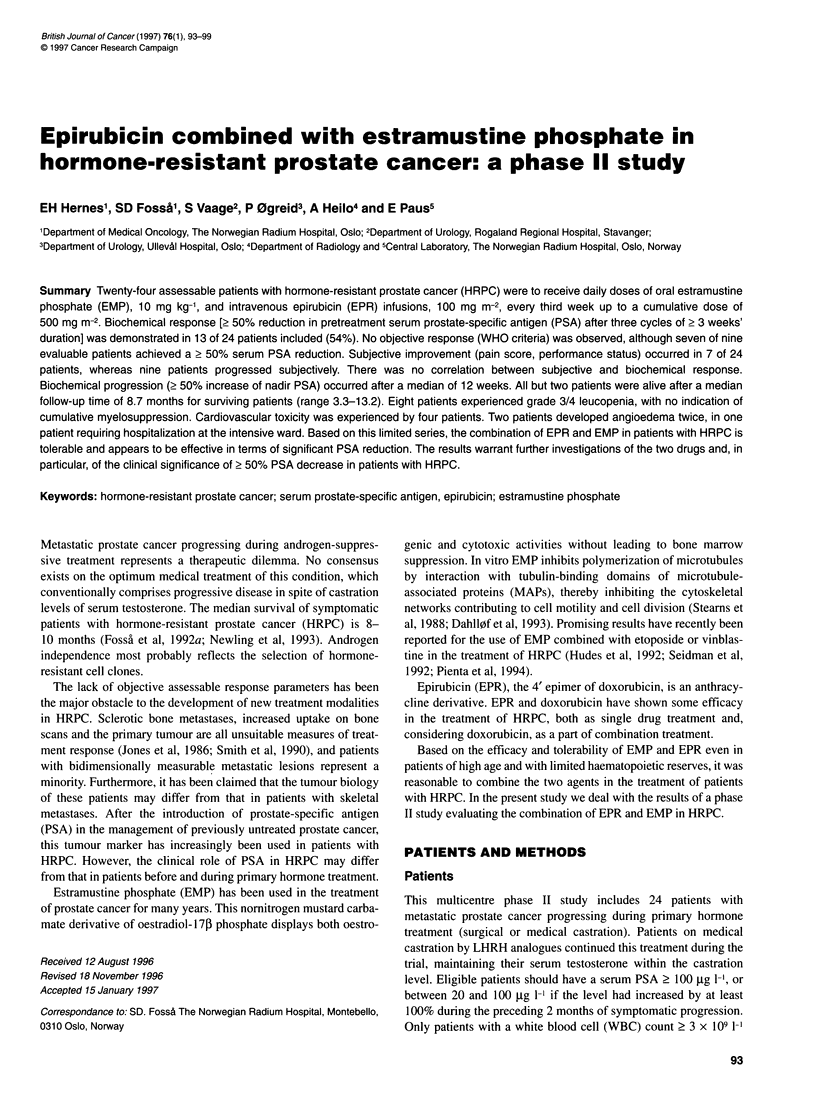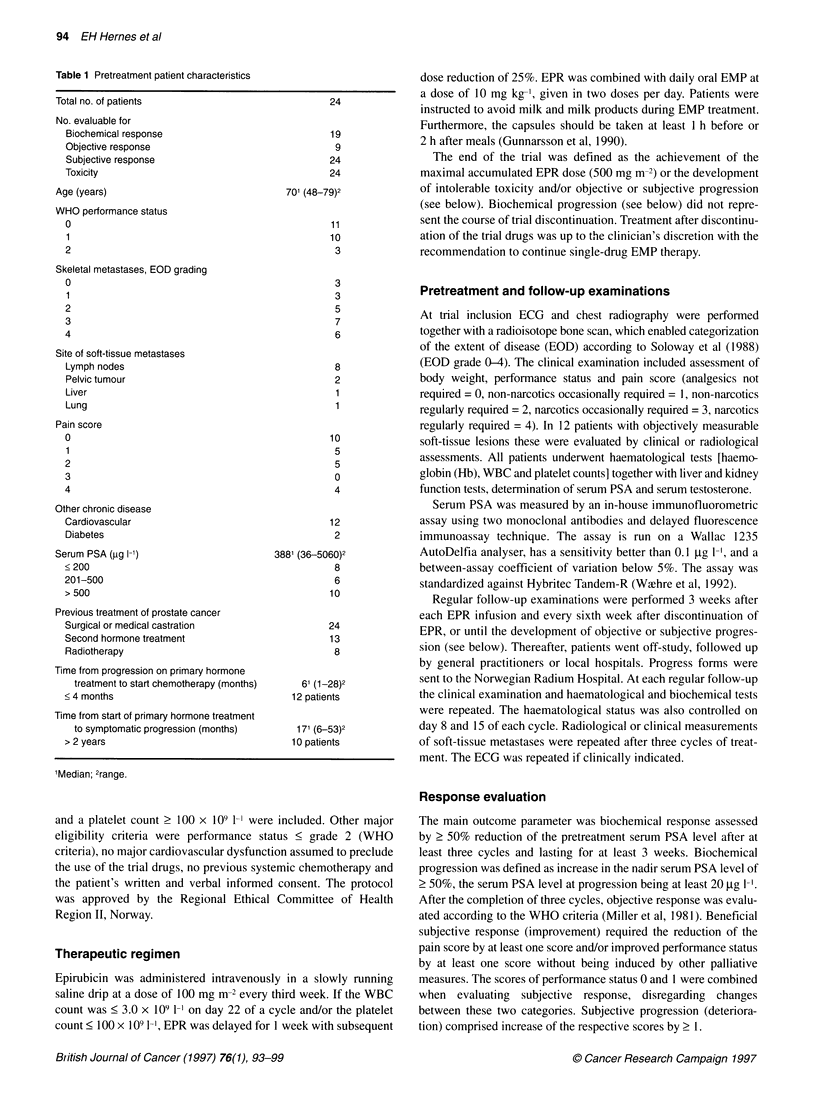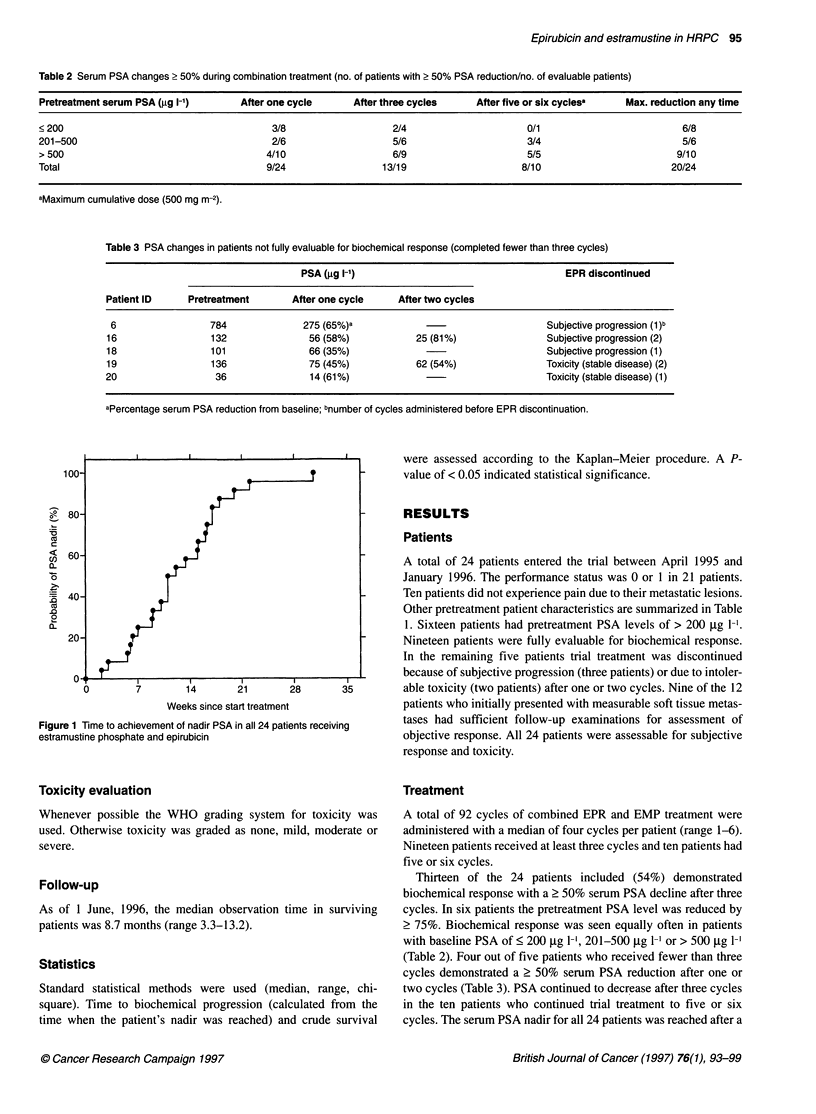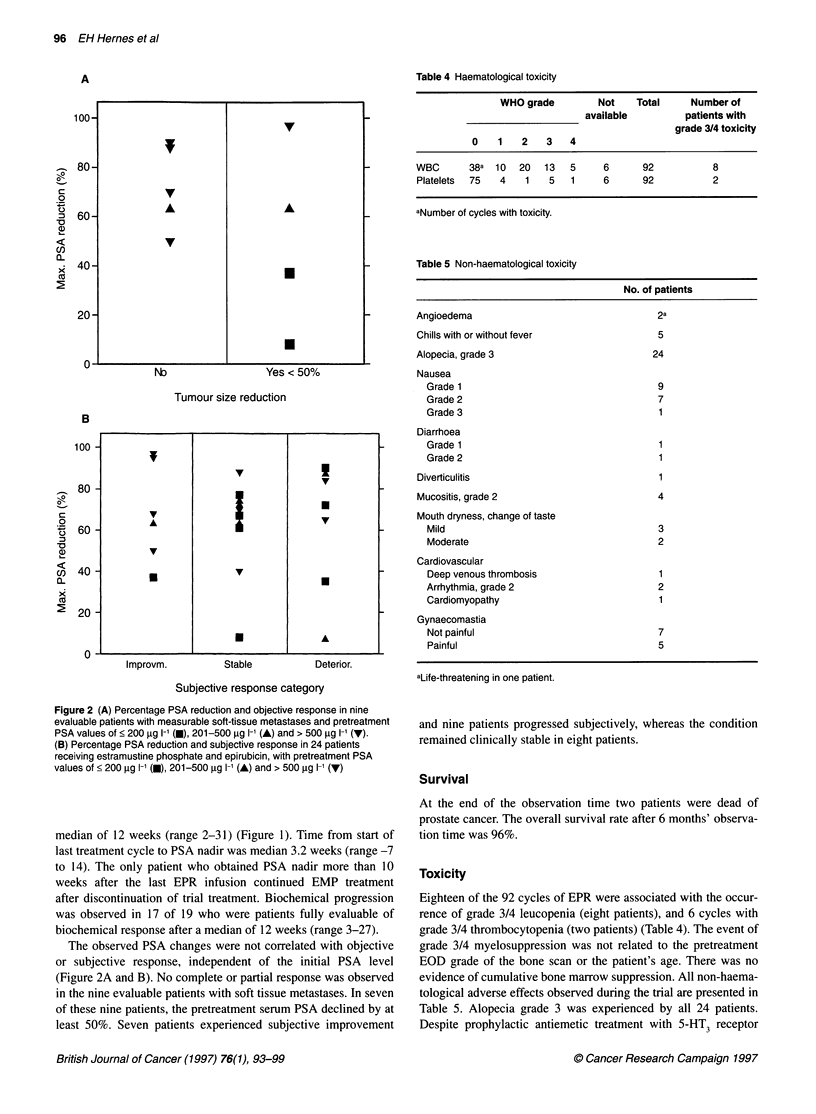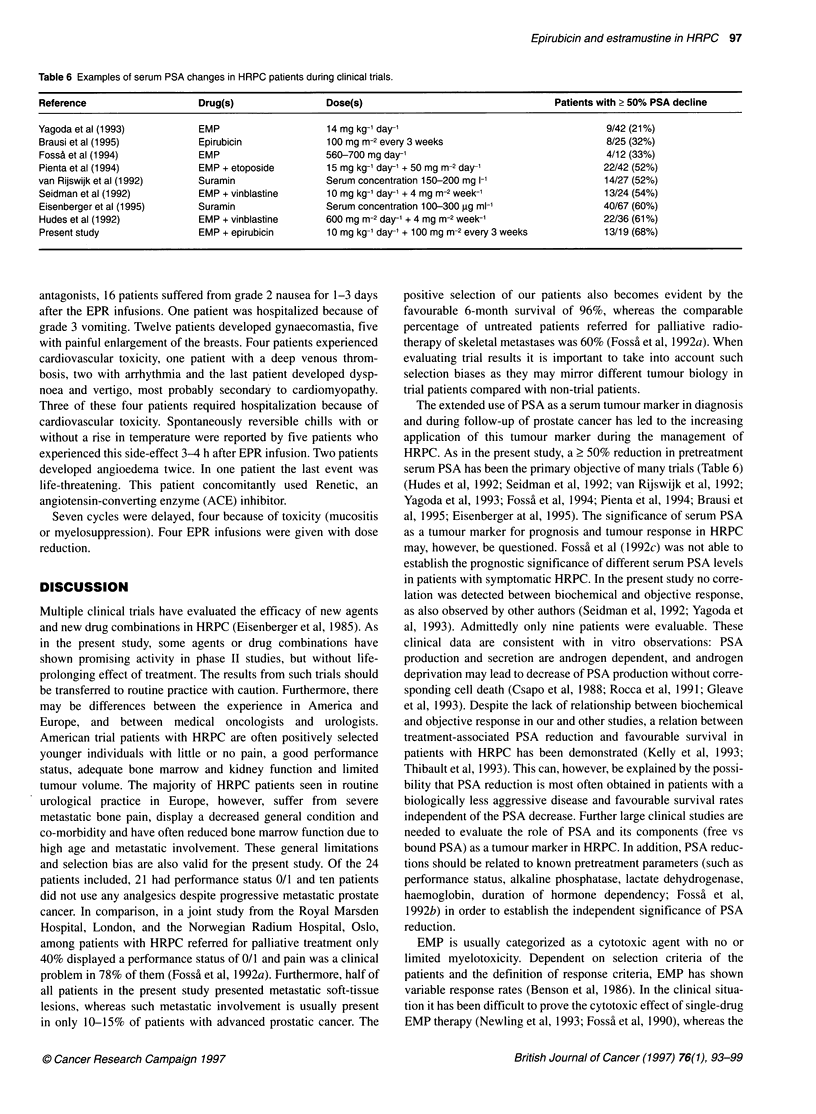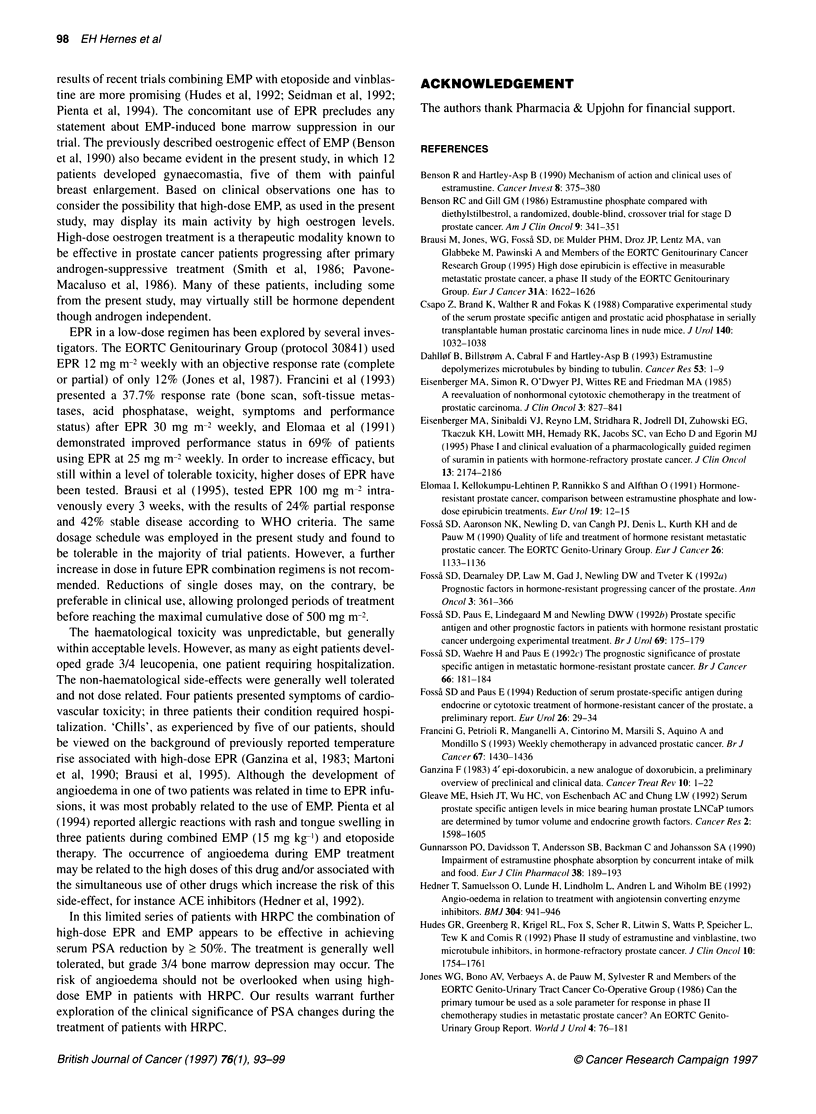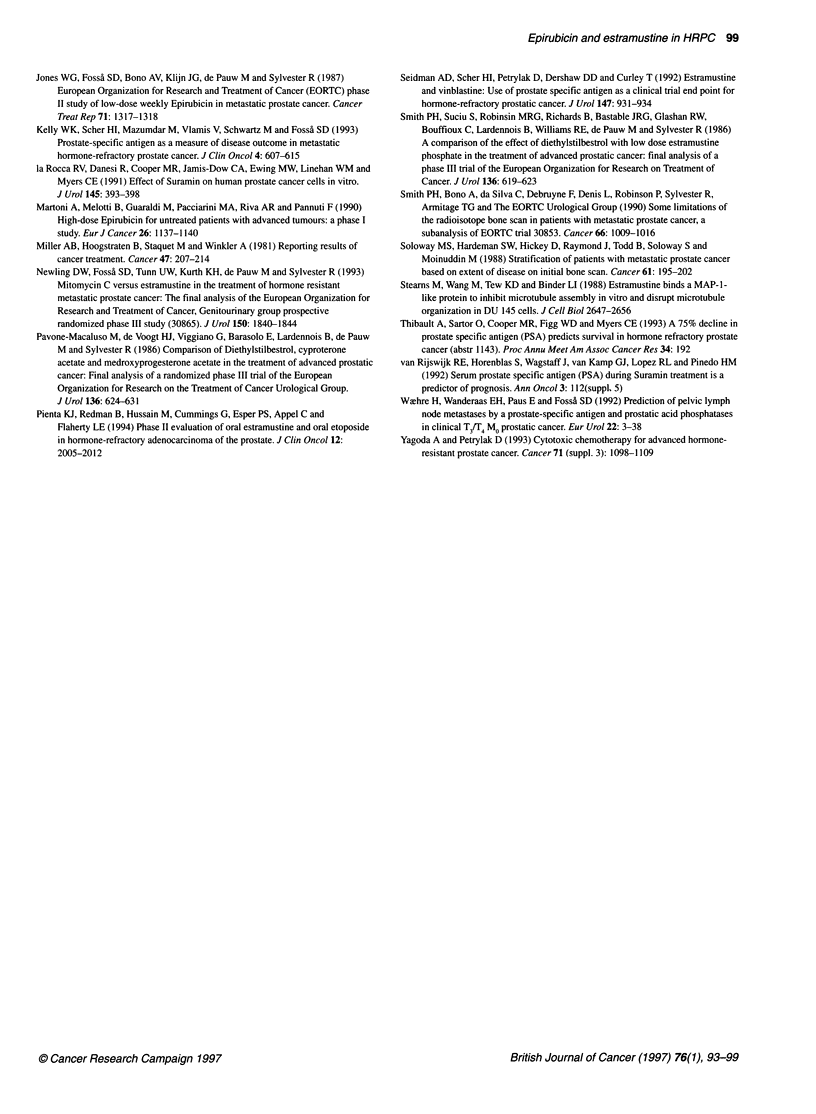# Epirubicin combined with estramustine phosphate in hormone-resistant prostate cancer: a phase II study.

**DOI:** 10.1038/bjc.1997.342

**Published:** 1997

**Authors:** E. H. Hernes, S. D. FossÃ¥, S. Vaage, P. Ogreid, A. Heilo, E. Paus

**Affiliations:** Department of Medical Oncology, The Norwegian Radium Hospital, Oslo, Norway.

## Abstract

Twenty-four assessable patients with hormone-resistant prostate cancer (HRPC) were to receive daily doses of oral estramustine phosphate (EMP), 10 mg kg(-1), and intravenous epirubicin (EPR) infusions, 100 mg m(-2), every third week up to a cumulative dose of 500 mg m(-2). Biochemical response [> or = 50% reduction in pretreatment serum prostate-specific antigen (PSA) after three cycles of > or = 3 weeks' duration] was demonstrated in 13 of 24 patients included (54%). No objective response (WHO criteria) was observed, although seven of nine evaluable patients achieved a > or = 50% serum PSA reduction. Subjective improvement (pain score, performance status) occurred in 7 of 24 patients, whereas nine patients progressed subjectively. There was no correlation between subjective and biochemical response. Biochemical progression (> or = 50% increase of nadir PSA) occurred after a median of 12 weeks. All but two patients were alive after a median follow-up time of 8.7 months for surviving patients (range 3.3-13.2). Eight patients experienced grade 3/4 leucopenia, with no indication of cumulative myelosuppression. Cardiovascular toxicity was experienced by four patients. Two patients developed angioedema twice, in one patient requiring hospitalization at the intensive ward. Based on this limited series, the combination of EPR and EMP in patients with HRPC is tolerable and appears to be effective in terms of significant PSA reduction. The results warrant further investigations of the two drugs and, in particular, of the clinical significance of > or = 50% PSA decrease in patients with HRPC.


British Journal of Cancer (1997) 76(1), 93–99
© 1997 Cancer Research Campaign

### Epirubicin combined with estramustine phosphate in hormone-resistant prostate cancer: a phase II study

**EH Hernes[1], SD Fosså[1], S Vaage[2], P Øgreid[3], A Heilo[4] and E Paus[5]**

[1]Department of Medical Oncology, The Norwegian Radium Hospital, Oslo; [2]Department of Urology, Rogaland Regional Hospital, Stavanger; [3]Department of Urology, Ullevål Hospital, Oslo; [4]Department of Radiology and [5]Central Laboratory, The Norwegian Radium Hospital, Oslo, Norway

**Summary** Twenty-four assessable patients with hormone-resistant prostate cancer (HRPC) were to receive daily doses of oral estramustine phosphate (EMP), 10 mg $kg^{-1}$, and intravenous epirubicin (EPR) infusions, 100 mg $m^{-2}$, every third week up to a cumulative dose of 500 mg $m^{-2}$. Biochemical response [≥ 50% reduction in pretreatment serum prostate-specific antigen (PSA) after three cycles of ≥ 3 weeks' duration] was demonstrated in 13 of 24 patients included (54%). No objective response (WHO criteria) was observed, although seven of nine evaluable patients achieved a ≥ 50% serum PSA reduction. Subjective improvement (pain score, performance status) occurred in 7 of 24 patients, whereas nine patients progressed subjectively. There was no correlation between subjective and biochemical response. Biochemical progression (≥ 50% increase of nadir PSA) occurred after a median of 12 weeks. All but two patients were alive after a median follow-up time of 8.7 months for surviving patients (range 3.3–13.2). Eight patients experienced grade 3/4 leucopenia, with no indication of cumulative myelosuppression. Cardiovascular toxicity was experienced by four patients. Two patients developed angioedema twice, in one patient requiring hospitalization at the intensive ward. Based on this limited series, the combination of EPR and EMP in patients with HRPC is tolerable and appears to be effective in terms of significant PSA reduction. The results warrant further investigations of the two drugs and, in particular, of the clinical significance of ≥ 50% PSA decrease in patients with HRPC.

**Keywords:** hormone-resistant prostate cancer; serum prostate-specific antigen, epirubicin; estramustine phosphate

Metastatic prostate cancer progressing during androgen-suppressive treatment represents a therapeutic dilemma. No consensus exists on the optimum medical treatment of this condition, which conventionally comprises progressive disease in spite of castration levels of serum testosterone. The median survival of symptomatic patients with hormone-resistant prostate cancer (HRPC) is 8–10 months (Fosså et al, 1992*a*; Newling et al, 1993). Androgen independence most probably reflects the selection of hormone-resistant cell clones.

The lack of objective assessable response parameters has been the major obstacle to the development of new treatment modalities in HRPC. Sclerotic bone metastases, increased uptake on bone scans and the primary tumour are all unsuitable measures of treatment response (Jones et al, 1986; Smith et al, 1990), and patients with bidimensionally measurable metastatic lesions represent a minority. Furthermore, it has been claimed that the tumour biology of these patients may differ from that in patients with skeletal metastases. After the introduction of prostate-specific antigen (PSA) in the management of previously untreated prostate cancer, this tumour marker has increasingly been used in patients with HRPC. However, the clinical role of PSA in HRPC may differ from that in patients before and during primary hormone treatment.

Estramustine phosphate (EMP) has been used in the treatment of prostate cancer for many years. This nornitrogen mustard carbamate derivative of oestradiol-17β phosphate displays both oestrogenic and cytotoxic activities without leading to bone marrow suppression. In vitro EMP inhibits polymerization of microtubules by interaction with tubulin-binding domains of microtubule-associated proteins (MAPs), thereby inhibiting the cytoskeletal networks contributing to cell motility and cell division (Stearns et al, 1988; Dahlløf et al, 1993). Promising results have recently been reported for the use of EMP combined with etoposide or vinblastine in the treatment of HRPC (Hudes et al, 1992; Seidman et al, 1992; Pienta et al, 1994).

Epirubicin (EPR), the 4′ epimer of doxorubicin, is an anthracycline derivative. EPR and doxorubicin have shown some efficacy in the treatment of HRPC, both as single drug treatment and, considering doxorubicin, as a part of combination treatment.

Based on the efficacy and tolerability of EMP and EPR even in patients of high age and with limited haematopoietic reserves, it was reasonable to combine the two agents in the treatment of patients with HRPC. In the present study we deal with the results of a phase II study evaluating the combination of EPR and EMP in HRPC.

### PATIENTS AND METHODS

### Patients

This multicentre phase II study includes 24 patients with metastatic prostate cancer progressing during primary hormone treatment (surgical or medical castration). Patients on medical castration by LHRH analogues continued this treatment during the trial, maintaining their serum testosterone within the castration level. Eligible patients should have a serum PSA ≥ 100 μg $l^{-1}$, or between 20 and 100 μg $l^{-1}$ if the level had increased by at least 100% during the preceding 2 months of symptomatic progression. Only patients with a white blood cell (WBC) count ≥ 3 × $10^9$ $l^{-1}$

*Received 12 August 1996*
*Revised 18 November 1996*
*Accepted 15 January 1997*

*Correspondance to:* SD. Fosså The Norwegian Radium Hospital, Montebello, 0310 Oslo, Norway

Table 1 Pretreatment patient characteristics

| | |
|---|---|
| Total no. of patients | 24 |
| No. evaluable for | |
| Biochemical response | 19 |
| Objective response | 9 |
| Subjective response | 24 |
| Toxicity | 24 |
| Age (years) | 70[1] (48–79)[2] |
| WHO performance status | |
| 0 | 11 |
| 1 | 10 |
| 2 | 3 |
| Skeletal metastases, EOD grading | |
| 0 | 3 |
| 1 | 3 |
| 2 | 5 |
| 3 | 7 |
| 4 | 6 |
| Site of soft-tissue metastases | |
| Lymph nodes | 8 |
| Pelvic tumour | 2 |
| Liver | 1 |
| Lung | 1 |
| Pain score | |
| 0 | 10 |
| 1 | 5 |
| 2 | 5 |
| 3 | 0 |
| 4 | 4 |
| Other chronic disease | |
| Cardiovascular | 12 |
| Diabetes | 2 |
| Serum PSA (μg $l^{-1}$) | 388[1] (36–5060)[2] |
| ≤ 200 | 8 |
| 201–500 | 6 |
| > 500 | 10 |
| Previous treatment of prostate cancer | |
| Surgical or medical castration | 24 |
| Second hormone treatment | 13 |
| Radiotherapy | 8 |
| Time from progression on primary hormone treatment to start chemotherapy (months) | 6[1] (1–28)[2] |
| ≤ 4 months | 12 patients |
| Time from start of primary hormone treatment to symptomatic progression (months) | 17[1] (6–53)[2] |
| > 2 years | 10 patients |

[1]Median; [2]range.

and a platelet count ≥ 100 × $10^9$ $l^{-1}$ were included. Other major eligibility criteria were performance status ≤ grade 2 (WHO criteria), no major cardiovascular dysfunction assumed to preclude the use of the trial drugs, no previous systemic chemotherapy and the patient's written and verbal informed consent. The protocol was approved by the Regional Ethical Committee of Health Region II, Norway.

### Therapeutic regimen

Epirubicin was administered intravenously in a slowly running saline drip at a dose of 100 mg $m^{-2}$ every third week. If the WBC count was ≤ 3.0 × $10^9$ $l^{-1}$ on day 22 of a cycle and/or the platelet count ≤ 100 × $10^9$ $l^{-1}$, EPR was delayed for 1 week with subsequent dose reduction of 25%. EPR was combined with daily oral EMP at a dose of 10 mg $kg^{-1}$, given in two doses per day. Patients were instructed to avoid milk and milk products during EMP treatment. Furthermore, the capsules should be taken at least 1 h before or 2 h after meals (Gunnarsson et al, 1990).

The end of the trial was defined as the achievement of the maximal accumulated EPR dose (500 mg $m^{-2}$) or the development of intolerable toxicity and/or objective or subjective progression (see below). Biochemical progression (see below) did not represent the course of trial discontinuation. Treatment after discontinuation of the trial drugs was up to the clinician's discretion with the recommendation to continue single-drug EMP therapy.

### Pretreatment and follow-up examinations

At trial inclusion ECG and chest radiography were performed together with a radioisotope bone scan, which enabled categorization of the extent of disease (EOD) according to Soloway et al (1988) (EOD grade 0–4). The clinical examination included assessment of body weight, performance status and pain score (analgesics not required = 0, non-narcotics occasionally required = 1, non-narcotics regularly required = 2, narcotics occasionally required = 3, narcotics regularly required = 4). In 12 patients with objectively measurable soft-tissue lesions these were evaluated by clinical or radiological assessments. All patients underwent haematological tests [haemoglobin (Hb), WBC and platelet counts] together with liver and kidney function tests, determination of serum PSA and serum testosterone.

Serum PSA was measured by an in-house immunofluorometric assay using two monoclonal antibodies and delayed fluorescence immunoassay technique. The assay is run on a Wallac 1235 AutoDelfia analyser, has a sensitivity better than 0.1 μg $l^{-1}$, and a between-assay coefficient of variation below 5%. The assay was standardized against Hybritec Tandem-R (Wæhre et al, 1992).

Regular follow-up examinations were performed 3 weeks after each EPR infusion and every sixth week after discontinuation of EPR, or until the development of objective or subjective progression (see below). Thereafter, patients went off-study, followed up by general practitioners or local hospitals. Progress forms were sent to the Norwegian Radium Hospital. At each regular follow-up the clinical examination and haematological and biochemical tests were repeated. The haematological status was also controlled on day 8 and 15 of each cycle. Radiological or clinical measurements of soft-tissue metastases were repeated after three cycles of treatment. The ECG was repeated if clinically indicated.

### Response evaluation

The main outcome parameter was biochemical response assessed by ≥ 50% reduction of the pretreatment serum PSA level after at least three cycles and lasting for at least 3 weeks. Biochemical progression was defined as increase in the nadir serum PSA level of ≥ 50%, the serum PSA level at progression being at least 20 μg $l^{-1}$. After the completion of three cycles, objective response was evaluated according to the WHO criteria (Miller et al, 1981). Beneficial subjective response (improvement) required the reduction of the pain score by at least one score and/or improved performance status by at least one score without being induced by other palliative measures. The scores of performance status 0 and 1 were combined when evaluating subjective response, disregarding changes between these two categories. Subjective progression (deterioration) comprised increase of the respective scores by ≥ 1.

© Cancer Research Campaign 1997

**Table 2** Serum PSA changes ≥ 50% during combination treatment (no. of patients with ≥ 50% PSA reduction/no. of evaluable patients)

| Pretreatment serum PSA (μg l$^{-1}$) | After one cycle | After three cycles | After five or six cycles[a] | Max. reduction any time |
|---|---|---|---|---|
| ≤ 200 | 3/8 | 2/4 | 0/1 | 6/8 |
| 201–500 | 2/6 | 5/6 | 3/4 | 5/6 |
| > 500 | 4/10 | 6/9 | 5/5 | 9/10 |
| Total | 9/24 | 13/19 | 8/10 | 20/24 |

[a]Maximum cumulative dose (500 mg m$^{-2}$).

**Table 3** PSA changes in patients not fully evaluable for biochemical response (completed fewer than three cycles)

| | PSA (μg l$^{-1}$) | | | EPR discontinued |
|---|---|---|---|---|
| Patient ID | Pretreatment | After one cycle | After two cycles | |
| 6 | 784 | 275 (65%)[a] | — | Subjective progression (1)[b] |
| 16 | 132 | 56 (58%) | 25 (81%) | Subjective progression (2) |
| 18 | 101 | 66 (35%) | — | Subjective progression (1) |
| 19 | 136 | 75 (45%) | 62 (54%) | Toxicity (stable disease) (2) |
| 20 | 36 | 14 (61%) | — | Toxicity (stable disease) (1) |

[a]Percentage serum PSA reduction from baseline; [b]number of cycles administered before EPR discontinuation.

**Figure 1** Time to achievement of nadir PSA in all 24 patients receiving estramustine phosphate and epirubicin

### Toxicity evaluation

Whenever possible the WHO grading system for toxicity was used. Otherwise toxicity was graded as none, mild, moderate or severe.

### Follow-up

As of 1 June, 1996, the median observation time in surviving patients was 8.7 months (range 3.3–13.2).

### Statistics

Standard statistical methods were used (median, range, chi-square). Time to biochemical progression (calculated from the time when the patient's nadir was reached) and crude survival were assessed according to the Kaplan–Meier procedure. A *P*-value of < 0.05 indicated statistical significance.

### RESULTS

### Patients

A total of 24 patients entered the trial between April 1995 and January 1996. The performance status was 0 or 1 in 21 patients. Ten patients did not experience pain due to their metastatic lesions. Other pretreatment patient characteristics are summarized in Table 1. Sixteen patients had pretreatment PSA levels of > 200 μg l$^{-1}$. Nineteen patients were fully evaluable for biochemical response. In the remaining five patients trial treatment was discontinued because of subjective progression (three patients) or due to intolerable toxicity (two patients) after one or two cycles. Nine of the 12 patients who initially presented with measurable soft tissue metastases had sufficient follow-up examinations for assessment of objective response. All 24 patients were assessable for subjective response and toxicity.

### Treatment

A total of 92 cycles of combined EPR and EMP treatment were administered with a median of four cycles per patient (range 1–6). Nineteen patients received at least three cycles and ten patients had five or six cycles.

Thirteen of the 24 patients included (54%) demonstrated biochemical response with a ≥ 50% serum PSA decline after three cycles. In six patients the pretreatment PSA level was reduced by ≥ 75%. Biochemical response was seen equally often in patients with baseline PSA of ≤ 200 μg l$^{-1}$, 201–500 μg l$^{-1}$ or > 500 μg l$^{-1}$ (Table 2). Four out of five patients who received fewer than three cycles demonstrated a ≥ 50% serum PSA reduction after one or two cycles (Table 3). PSA continued to decrease after three cycles in the ten patients who continued trial treatment to five or six cycles. The serum PSA nadir for all 24 patients was reached after a

© Cancer Research Campaign 1997 

**Figure 2** (**A**) Percentage PSA reduction and objective response in nine evaluable patients with measurable soft-tissue metastases and pretreatment PSA values of ≤ 200 μg l⁻¹ (■), 201–500 μg l⁻¹ (▲) and > 500 μg l⁻¹ (▼). (**B**) Percentage PSA reduction and subjective response in 24 patients receiving estramustine phosphate and epirubicin, with pretreatment PSA values of ≤ 200 μg l⁻¹ (■), 201–500 μg l⁻¹ (▲) and > 500 μg l⁻¹ (▼)

median of 12 weeks (range 2–31) (Figure 1). Time from start of last treatment cycle to PSA nadir was median 3.2 weeks (range –7 to 14). The only patient who obtained PSA nadir more than 10 weeks after the last EPR infusion continued EMP treatment after discontinuation of trial treatment. Biochemical progression was observed in 17 of 19 who were patients fully evaluable of biochemical response after a median of 12 weeks (range 3–27).

The observed PSA changes were not correlated with objective or subjective response, independent of the initial PSA level (Figure 2A and B). No complete or partial response was observed in the nine evaluable patients with soft tissue metastases. In seven of these nine patients, the pretreatment serum PSA declined by at least 50%. Seven patients experienced subjective improvement

**Table 4** Haematological toxicity

| | WHO grade | | | | | Not available | Total | Number of patients with grade 3/4 toxicity |
|---|---|---|---|---|---|---|---|---|
| | 0 | 1 | 2 | 3 | 4 | | | |
| WBC | 38[a] | 10 | 20 | 13 | 5 | 6 | 92 | 8 |
| Platelets | 75 | 4 | 1 | 5 | 1 | 6 | 92 | 2 |

[a]Number of cycles with toxicity.

**Table 5** Non-haematological toxicity

| | No. of patients |
|---|---|
| Angioedema | 2[a] |
| Chills with or without fever | 5 |
| Alopecia, grade 3 | 24 |
| Nausea | |
| Grade 1 | 9 |
| Grade 2 | 7 |
| Grade 3 | 1 |
| Diarrhoea | |
| Grade 1 | 1 |
| Grade 2 | 1 |
| Diverticulitis | 1 |
| Mucositis, grade 2 | 4 |
| Mouth dryness, change of taste | |
| Mild | 3 |
| Moderate | 2 |
| Cardiovascular | |
| Deep venous thrombosis | 1 |
| Arrhythmia, grade 2 | 2 |
| Cardiomyopathy | 1 |
| Gynaecomastia | |
| Not painful | 7 |
| Painful | 5 |

[a]Life-threatening in one patient.

and nine patients progressed subjectively, whereas the condition remained clinically stable in eight patients.

### Survival

At the end of the observation time two patients were dead of prostate cancer. The overall survival rate after 6 months' observation time was 96%.

### Toxicity

Eighteen of the 92 cycles of EPR were associated with the occurrence of grade 3/4 leucopenia (eight patients), and 6 cycles with grade 3/4 thrombocytopenia (two patients) (Table 4). The event of grade 3/4 myelosuppression was not related to the pretreatment EOD grade of the bone scan or the patient's age. There was no evidence of cumulative bone marrow suppression. All non-haematological adverse effects observed during the trial are presented in Table 5. Alopecia grade 3 was experienced by all 24 patients. Despite prophylactic antiemetic treatment with 5-$HT_3$ receptor

© Cancer Research Campaign 1997

**Table 6** Examples of serum PSA changes in HRPC patients during clinical trials.

| Reference | Drug(s) | Dose(s) | Patients with ≥ 50% PSA decline |
|---|---|---|---|
| Yagoda et al (1993) | EMP | 14 mg $kg^{-1}$ $day^{-1}$ | 9/42 (21%) |
| Brausi et al (1995) | Epirubicin | 100 mg $m^{-2}$ every 3 weeks | 8/25 (32%) |
| Fosså et al (1994) | EMP | 560–700 mg $day^{-1}$ | 4/12 (33%) |
| Pienta et al (1994) | EMP + etoposide | 15 mg $kg^{-1}$ $day^{-1}$ + 50 mg $m^{-2}$ $day^{-1}$ | 22/42 (52%) |
| van Rijswijk et al (1992) | Suramin | Serum concentration 150–200 mg $l^{-1}$ | 14/27 (52%) |
| Seidman et al (1992) | EMP + vinblastine | 10 mg $kg^{-1}$ $day^{-1}$ + 4 mg $m^{-2}$ $week^{-1}$ | 13/24 (54%) |
| Eisenberger et al (1995) | Suramin | Serum concentration 100–300 μg $ml^{-1}$ | 40/67 (60%) |
| Hudes et al (1992) | EMP + vinblastine | 600 mg $m^{-2}$ $day^{-1}$ + 4 mg $m^{-2}$ $week^{-1}$ | 22/36 (61%) |
| Present study | EMP + epirubicin | 10 mg $kg^{-1}$ $day^{-1}$ + 100 mg $m^{-2}$ every 3 weeks | 13/19 (68%) |

antagonists, 16 patients suffered from grade 2 nausea for 1–3 days after the EPR infusions. One patient was hospitalized because of grade 3 vomiting. Twelve patients developed gynaecomastia, five with painful enlargement of the breasts. Four patients experienced cardiovascular toxicity, one patient with a deep venous thrombosis, two with arrhythmia and the last patient developed dyspnoea and vertigo, most probably secondary to cardiomyopathy. Three of these four patients required hospitalization because of cardiovascular toxicity. Spontaneously reversible chills with or without a rise in temperature were reported by five patients who experienced this side-effect 3–4 h after EPR infusion. Two patients developed angioedema twice. In one patient the last event was life-threatening. This patient concomitantly used Renetic, an angiotensin-converting enzyme (ACE) inhibitor.

Seven cycles were delayed, four because of toxicity (mucositis or myelosuppression). Four EPR infusions were given with dose reduction.

### DISCUSSION

Multiple clinical trials have evaluated the efficacy of new agents and new drug combinations in HRPC (Eisenberger et al, 1985). As in the present study, some agents or drug combinations have shown promising activity in phase II studies, but without life-prolonging effect of treatment. The results from such trials should be transferred to routine practice with caution. Furthermore, there may be differences between the experience in America and Europe, and between medical oncologists and urologists. American trial patients with HRPC are often positively selected younger individuals with little or no pain, a good performance status, adequate bone marrow and kidney function and limited tumour volume. The majority of HRPC patients seen in routine urological practice in Europe, however, suffer from severe metastatic bone pain, display a decreased general condition and co-morbidity and have often reduced bone marrow function due to high age and metastatic involvement. These general limitations and selection bias are also valid for the present study. Of the 24 patients included, 21 had performance status 0/1 and ten patients did not use any analgesics despite progressive metastatic prostate cancer. In comparison, in a joint study from the Royal Marsden Hospital, London, and the Norwegian Radium Hospital, Oslo, among patients with HRPC referred for palliative treatment only 40% displayed a performance status of 0/1 and pain was a clinical problem in 78% of them (Fosså et al, 1992*a*). Furthermore, half of all patients in the present study presented metastatic soft-tissue lesions, whereas such metastatic involvement is usually present in only 10–15% of patients with advanced prostatic cancer. The positive selection of our patients also becomes evident by the favourable 6-month survival of 96%, whereas the comparable percentage of untreated patients referred for palliative radiotherapy of skeletal metastases was 60% (Fosså et al, 1992*a*). When evaluating trial results it is important to take into account such selection biases as they may mirror different tumour biology in trial patients compared with non-trial patients.

The extended use of PSA as a serum tumour marker in diagnosis and during follow-up of prostate cancer has led to the increasing application of this tumour marker during the management of HRPC. As in the present study, a ≥ 50% reduction in pretreatment serum PSA has been the primary objective of many trials (Table 6) (Hudes et al, 1992; Seidman et al, 1992; van Rijswijk et al, 1992; Yagoda et al, 1993; Fosså et al, 1994; Pienta et al, 1994; Brausi et al, 1995; Eisenberger at al, 1995). The significance of serum PSA as a tumour marker for prognosis and tumour response in HRPC may, however, be questioned. Fosså et al (1992*c*) was not able to establish the prognostic significance of different serum PSA levels in patients with symptomatic HRPC. In the present study no correlation was detected between biochemical and objective response, as also observed by other authors (Seidman et al, 1992; Yagoda et al, 1993). Admittedly only nine patients were evaluable. These clinical data are consistent with in vitro observations: PSA production and secretion are androgen dependent, and androgen deprivation may lead to decrease of PSA production without corresponding cell death (Csapo et al, 1988; Rocca et al, 1991; Gleave et al, 1993). Despite the lack of relationship between biochemical and objective response in our and other studies, a relation between treatment-associated PSA reduction and favourable survival in patients with HRPC has been demonstrated (Kelly et al, 1993; Thibault et al, 1993). This can, however, be explained by the possibility that PSA reduction is most often obtained in patients with a biologically less aggressive disease and favourable survival rates independent of the PSA decrease. Further large clinical studies are needed to evaluate the role of PSA and its components (free vs bound PSA) as a tumour marker in HRPC. In addition, PSA reductions should be related to known pretreatment parameters (such as performance status, alkaline phosphatase, lactate dehydrogenase, haemoglobin, duration of hormone dependency; Fosså et al, 1992*b*) in order to establish the independent significance of PSA reduction.

EMP is usually categorized as a cytotoxic agent with no or limited myelotoxicity. Dependent on selection criteria of the patients and the definition of response criteria, EMP has shown variable response rates (Benson et al, 1986). In the clinical situation it has been difficult to prove the cytotoxic effect of single-drug EMP therapy (Newling et al, 1993; Fosså et al, 1990), whereas the

© Cancer Research Campaign 1997 

results of recent trials combining EMP with etoposide and vinblastine are more promising (Hudes et al, 1992; Seidman et al, 1992; Pienta et al, 1994). The concomitant use of EPR precludes any statement about EMP-induced bone marrow suppression in our trial. The previously described oestrogenic effect of EMP (Benson et al, 1990) also became evident in the present study, in which 12 patients developed gynaecomastia, five of them with painful breast enlargement. Based on clinical observations one has to consider the possibility that high-dose EMP, as used in the present study, may display its main activity by high oestrogen levels. High-dose oestrogen treatment is a therapeutic modality known to be effective in prostate cancer patients progressing after primary androgen-suppressive treatment (Smith et al, 1986; Pavone-Macaluso et al, 1986). Many of these patients, including some from the present study, may virtually still be hormone dependent though androgen independent.

EPR in a low-dose regimen has been explored by several investigators. The EORTC Genitourinary Group (protocol 30841) used EPR 12 mg $m^{-2}$ weekly with an objective response rate (complete or partial) of only 12% (Jones et al, 1987). Francini et al (1993) presented a 37.7% response rate (bone scan, soft-tissue metastases, acid phosphatase, weight, symptoms and performance status) after EPR 30 mg $m^{-2}$ weekly, and Elomaa et al (1991) demonstrated improved performance status in 69% of patients using EPR at 25 mg $m^{-2}$ weekly. In order to increase efficacy, but still within a level of tolerable toxicity, higher doses of EPR have been tested. Brausi et al (1995), tested EPR 100 mg $m^{-2}$ intravenously every 3 weeks, with the results of 24% partial response and 42% stable disease according to WHO criteria. The same dosage schedule was employed in the present study and found to be tolerable in the majority of trial patients. However, a further increase in dose in future EPR combination regimens is not recommended. Reductions of single doses may, on the contrary, be preferable in clinical use, allowing prolonged periods of treatment before reaching the maximal cumulative dose of 500 mg $m^{-2}$.

The haematological toxicity was unpredictable, but generally within acceptable levels. However, as many as eight patients developed grade 3/4 leucopenia, one patient requiring hospitalization. The non-haematological side-effects were generally well tolerated and not dose related. Four patients presented symptoms of cardiovascular toxicity; in three patients their condition required hospitalization. 'Chills', as experienced by five of our patients, should be viewed on the background of previously reported temperature rise associated with high-dose EPR (Ganzina et al, 1983; Martoni et al, 1990; Brausi et al, 1995). Although the development of angioedema in one of two patients was related in time to EPR infusions, it was most probably related to the use of EMP. Pienta et al (1994) reported allergic reactions with rash and tongue swelling in three patients during combined EMP (15 mg $kg^{-1}$) and etoposide therapy. The occurrence of angioedema during EMP treatment may be related to the high doses of this drug and/or associated with the simultaneous use of other drugs which increase the risk of this side-effect, for instance ACE inhibitors (Hedner et al, 1992).

In this limited series of patients with HRPC the combination of high-dose EPR and EMP appears to be effective in achieving serum PSA reduction by $\geq$ 50%. The treatment is generally well tolerated, but grade 3/4 bone marrow depression may occur. The risk of angioedema should not be overlooked when using high-dose EMP in patients with HRPC. Our results warrant further exploration of the clinical significance of PSA changes during the treatment of patients with HRPC.

### ACKNOWLEDGEMENT

The authors thank Pharmacia & Upjohn for financial support.

### REFERENCES

Benson R and Hartley-Asp B (1990) Mechanism of action and clinical uses of estramustine. *Cancer Invest* **8**: 375–380

Benson RC and Gill GM (1986) Estramustine phosphate compared with diethylstilbestrol, a randomized, double-blind, crossover trial for stage D prostate cancer. *Am J Clin Oncol* **9**: 341–351

Brausi M, Jones, WG, Fosså SD, de Mulder PHM, Droz JP, Lentz MA, van Glabbeke M, Pawinski A and Members of the EORTC Genitourinary Cancer Research Group (1995) High dose epirubicin is effective in measurable metastatic prostate cancer, a phase II study of the EORTC Genitourinary Group. *Eur J Cancer* **31A**: 1622–1626

Csapo Z, Brand K, Walther R and Fokas K (1988) Comparative experimental study of the serum prostate specific antigen and prostatic acid phosphatase in serially transplantable human prostatic carcinoma lines in nude mice. *J Urol* **140**: 1032–1038

Dahllöf B, Billstrøm A, Cabral F and Hartley-Asp B (1993) Estramustine depolymerizes microtubules by binding to tubulin. *Cancer Res* **53**: 1–9

Eisenberger MA, Simon R, O'Dwyer PJ, Wittes RE and Friedman MA (1985) A reevaluation of nonhormonal cytotoxic chemotherapy in the treatment of prostatic carcinoma. *J Clin Oncol* **3**: 827–841

Eisenberger MA, Sinibaldi VJ, Reyno LM, Stridhara R, Jodrell DI, Zuhowski EG, Tkaczuk KH, Lowitt MH, Hemady RK, Jacobs SC, van Echo D and Egorin MJ (1995) Phase I and clinical evaluation of a pharmacologically guided regimen of suramin in patients with hormone-refractory prostate cancer. *J Clin Oncol* **13**: 2174–2186

Elomaa I, Kellokumpu-Lehtinen P, Rannikko S and Alfthan O (1991) Hormone-resistant prostate cancer, comparison between estramustine phosphate and low-dose epirubicin treatments. *Eur Urol* **19**: 12–15

Fosså SD, Aaronson NK, Newling D, van Cangh PJ, Denis L, Kurth KH and de Pauw M (1990) Quality of life and treatment of hormone resistant metastatic prostatic cancer. The EORTC Genito-Urinary Group. *Eur J Cancer* **26**: 1133–1136

Fosså SD, Dearnaley DP, Law M, Gad J, Newling DW and Tveter K (1992*a*) Prognostic factors in hormone-resistant progressing cancer of the prostate. *Ann Oncol* **3**: 361–366

Fosså SD, Paus E, Lindegaard M and Newling DWW (1992*b*) Prostate specific antigen and other prognostic factors in patients with hormone resistant prostatic cancer undergoing experimental treatment. *Br J Urol* **69**: 175–179

Fosså SD, Waehre H and Paus E (1992*c*) The prognostic significance of prostate specific antigen in metastatic hormone-resistant prostate cancer. *Br J Cancer* **66**: 181–184

Fosså SD and Paus E (1994) Reduction of serum prostate-specific antigen during endocrine or cytotoxic treatment of hormone-resistant cancer of the prostate, a preliminary report. *Eur Urol* **26**: 29–34

Francini G, Petrioli R, Manganelli A, Cintorino M, Marsili S, Aquino A and Mondillo S (1993) Weekly chemotherapy in advanced prostatic cancer. *Br J Cancer* **67**: 1430–1436

Ganzina F (1983) 4′ epi-doxorubicin, a new analogue of doxorubicin, a preliminary overview of preclinical and clinical data. *Cancer Treat Rev* **10**: 1–22

Gleave ME, Hsieh JT, Wu HC, von Eschenbach AC and Chung LW (1992) Serum prostate specific antigen levels in mice bearing human prostate LNCaP tumors are determined by tumor volume and endocrine growth factors. *Cancer Res* **2**: 1598–1605

Gunnarsson PO, Davidsson T, Andersson SB, Backman C and Johansson SA (1990) Impairment of estramustine phosphate absorption by concurrent intake of milk and food. *Eur J Clin Pharmacol* **38**: 189–193

Hedner T, Samuelsson O, Lunde H, Lindholm L, Andren L and Wiholm BE (1992) Angio-oedema in relation to treatment with angiotensin converting enzyme inhibitors. *BMJ* **304**: 941–946

Hudes GR, Greenberg R, Krigel RL, Fox S, Scher R, Litwin S, Watts P, Speicher L, Tew K and Comis R (1992) Phase II study of estramustine and vinblastine, two microtubule inhibitors, in hormone-refractory prostate cancer. *J Clin Oncol* **10**: 1754–1761

Jones WG, Bono AV, Verbaeys A, de Pauw M, Sylvester R and Members of the EORTC Genito-Urinary Tract Cancer Co-Operative Group (1986) Can the primary tumour be used as a sole parameter for response in phase II chemotherapy studies in metastatic prostate cancer? An EORTC Genito-Urinary Group Report. *World J Urol* **4**: 76–181

© Cancer Research Campaign 1997

Jones WG, Fosså SD, Bono AV, Klijn JG, de Pauw M and Sylvester R (1987) European Organization for Research and Treatment of Cancer (EORTC) phase II study of low-dose weekly Epirubicin in metastatic prostate cancer. *Cancer Treat Rep* **71**: 1317–1318

Kelly WK, Scher HI, Mazumdar M, Vlamis V, Schwartz M and Fosså SD (1993) Prostate-specific antigen as a measure of disease outcome in metastatic hormone-refractory prostate cancer. *J Clin Oncol* **4**: 607–615

la Rocca RV, Danesi R, Cooper MR, Jamis-Dow CA, Ewing MW, Linehan WM and Myers CE (1991) Effect of Suramin on human prostate cancer cells in vitro. *J Urol* **145**: 393–398

Martoni A, Melotti B, Guaraldi M, Pacciarini MA, Riva AR and Pannuti F (1990) High-dose Epirubicin for untreated patients with advanced tumours: a phase I study. *Eur J Cancer* **26**: 1137–1140

Miller AB, Hoogstraten B, Staquet M and Winkler A (1981) Reporting results of cancer treatment. *Cancer* **47**: 207–214

Newling DW, Fosså SD, Tunn UW, Kurth KH, de Pauw M and Sylvester R (1993) Mitomycin C versus estramustine in the treatment of hormone resistant metastatic prostate cancer: The final analysis of the European Organization for Research and Treatment of Cancer, Genitourinary group prospective randomized phase III study (30865). *J Urol* **150**: 1840–1844

Pavone-Macaluso M, de Voogt HJ, Viggiano G, Barasolo E, Lardennois B, de Pauw M and Sylvester R (1986) Comparison of Diethylstilbestrol, cyproterone acetate and medroxyprogesterone acetate in the treatment of advanced prostatic cancer: Final analysis of a randomized phase III trial of the European Organization for Research on the Treatment of Cancer Urological Group. *J Urol* **136**: 624–631

Pienta KJ, Redman B, Hussain M, Cummings G, Esper PS, Appel C and Flaherty LE (1994) Phase II evaluation of oral estramustine and oral etoposide in hormone-refractory adenocarcinoma of the prostate. *J Clin Oncol* **12**: 2005–2012

Seidman AD, Scher HI, Petrylak D, Dershaw DD and Curley T (1992) Estramustine and vinblastine: Use of prostate specific antigen as a clinical trial end point for hormone-refractory prostatic cancer. *J Urol* **147**: 931–934

Smith PH, Suciu S, Robinsin MRG, Richards B, Bastable JRG, Glashan RW, Bouffioux C, Lardennois B, Williams RE, de Pauw M and Sylvester R (1986) A comparison of the effect of diethylstilbestrol with low dose estramustine phosphate in the treatment of advanced prostatic cancer: final analysis of a phase III trial of the European Organization for Research on Treatment of Cancer. *J Urol* **136**: 619–623

Smith PH, Bono A, da Silva C, Debruyne F, Denis L, Robinson P, Sylvester R, Armitage TG and The EORTC Urological Group (1990) Some limitations of the radioisotope bone scan in patients with metastatic prostate cancer, a subanalysis of EORTC trial 30853. *Cancer* **66**: 1009–1016

Soloway MS, Hardeman SW, Hickey D, Raymond J, Todd B, Soloway S and Moinuddin M (1988) Stratification of patients with metastatic prostate cancer based on extent of disease on initial bone scan. *Cancer* **61**: 195–202

Stearns M, Wang M, Tew KD and Binder LI (1988) Estramustine binds a MAP-1-like protein to inhibit microtubule assembly in vitro and disrupt microtubule organization in DU 145 cells. *J Cell Biol* 2647–2656

Thibault A, Sartor O, Cooper MR, Figg WD and Myers CE (1993) A 75% decline in prostate specific antigen (PSA) predicts survival in hormone refractory prostate cancer (abstr 1143). *Proc Annu Meet Am Assoc Cancer Res* **34**: 192

van Rijswijk RE, Horenblas S, Wagstaff J, van Kamp GJ, Lopez RL and Pinedo HM (1992) Serum prostate specific antigen (PSA) during Suramin treatment is a predictor of prognosis. *Ann Oncol* **3**: 112(suppl. 5)

Wæhre H, Wanderaas EH, Paus E and Fosså SD (1992) Prediction of pelvic lymph node metastases by a prostate-specific antigen and prostatic acid phosphatases in clinical $T_3/T_4$ $M_0$ prostatic cancer. *Eur Urol* **22**: 3–38

Yagoda A and Petrylak D (1993) Cytotoxic chemotherapy for advanced hormone-resistant prostate cancer. *Cancer* **71** (suppl. 3): 1098–1109

© Cancer Research Campaign 1997